# Prognostic Markers for Chorioamnionitis: IL-6, TNF-α, and MMP-8 in Vaginally Obtained Amniotic Fluid

**DOI:** 10.3390/jcm10051136

**Published:** 2021-03-08

**Authors:** Greta Balciuniene, Violeta Gulbiniene, Irena Dumalakiene, Rita Viliene, Daiva Bartkeviciene, Ingrida Pilypiene, Grazina S. Drasutiene, Diana Ramasauskaite

**Affiliations:** 1 Clinic of Obstetrics, and Gynaecology, Institute of Clinical Medicine of the Faculty of Medicine of Vilnius University, 03101 Vilnius, Lithuania; greta.balciuniene@gmail.com (G.B.); violeta.gulbiniene@gmail.com (V.G.); daivabartk@gmail.com (D.B.); grazina.drasutiene@mf.vu.lt (G.S.D.); 2 Center of Obstetrics and Gynaecology, Vilnius University Hospital Santaros Klinikos, 08406 Vilnius, Lithuania; ingrida.pilypiene@gmail.com; 3 Department of Immunology, State Research Institute Centre for Innovative Medicine, 08410 Vilnius, Lithuania; irenadumalakiene@imcentras.lt (I.D.); ritviliene@gmail.com (R.V.)

**Keywords:** chorioamnionitis, immunological markers, IL-6, TNF-α, MMP-8

## Abstract

Background. Earlier chorioamnionitis diagnosis is crucial to improve maternal and neonatal health outcomes. This study was conducted to evaluate the inlerleukin-6 (IL-6), tumor necrosis factor α (TNF-α), and matrix metalloproteinase 8 (MMP-8) levels in vaginally obtained amniotic fluid to investigate their prognostic value and to determine the most appropriate cut-off values for the prediction of chorioamnionitis. Methods. This case control study included women who were diagnosed with preterm premature rupture of the membranes before 34 weeks of gestation and were admitted to Vilnius University Hospital Santaros Klinikos. Free-leaking amniotic fluid was obtained vaginally with a sterile speculum less than 48h before delivery. Amniotic fluid IL-6, TNF-α, and MMP-8 levels were determined by the Enzyme Linked Immunosorbent Assay. Diagnosis of chorioamnionitis was confirmed by histological examination of the placenta and membranes after delivery. Results. The study included 156 women, 65 patients in the histological chorioamnionitis group (Group I) and 91 in a group without diagnosed histological chorioamnionitis (Group II). The median concentrations of IL-6, MMP-8, and TNF-α in amniotic fluid were statistically significantly higher in Group I than in Group II (*p*-value < 0.001). The area under the curve of TNF-α and MMP-8 were higher than the area under the curve of IL-6 (0.91, 0.89, and 0.81, respectively). No statistically significant difference was found when comparing the receiver operating characteristic (ROC) curves of TNF-α and MMP-8. The optimum cut-off values for the prediction of chorioamnionitis were found to be 1389.82 pg/mL for IL-6, 21.17 pg/mL for TNF-α, and 172.53 ng/mL for MMP-8. The sensitivity, specificity, positive prognostic value (PPV), and negative prognostic value (NPV) of the IL-6 cut-off for chorioamnionitis were 88%, 70%, 67%, and 89%, respectively. The sensitivity, specificity, PPV, and NPV of the TNF-α cut-off were 88%, 84%, 79%, and 90%, respectively. The sensitivity, specificity, PPV, and NPV of the MMP-8 cut-off were 80%, 87%, 81%, and 86%, respectively. Conclusions. The vaginally obtained amniotic fluid IL-6, MMP-8, and TNF-α seem to be good predictors for chorioamnionitis of patients with preterm premature rupture of membranes before 34 weeks of gestation. The noninvasive technique of sampling amniotic fluid could be alternative method to invasive amniocentesis.

## 1. Introduction

Chorioamnionitis is a type of intra-amniotic infection that typically occurs due to an ascending polymicrobial bacterial infection in the setting of membrane rupture [1]. It is a complication of up to 60% of preterm premature rupture of membrane (PPROM) cases [2]. Chorioamnionitis leads to fetal inflammatory response syndrome, which is associated with higher rates of neonatal morbidity (cerebral palsy, intracranial hemorrhage, sepsis, respiratory distress syndrome, necrotizing enterocolitis, and neurodevelopmental disorders) and mortality [3,4].

Histological, microbiological, biochemical, and clinical criteria can be used to define chorioamnionitis. A diagnosis of clinical chorioamnionitis is based on the presence of an elevated maternal body temperature to 37.8 ℃ and presentation with at least two of the following criteria: uterine tenderness, foul-smelling vaginal discharge, maternal leukocytosis (>15,000 cells/mm^3^), maternal tachycardia (>100 beats/min), and/or fetal tachycardia (>160 beats/min) [5]. These Gibbs criteria were introduced more than 40 years ago and are limited because of their low sensitivity and specificity [6,7,8]. Histological examination of the placenta is considered the gold standard to diagnose intrauterine infection, but this finding is only available after delivery [9]. Some studies have demonstrated that only 5–10% of histological chorioamnionitis cases are diagnosed clinically [9,10]. 

Many studies have been conducted to identify amniotic fluid markers to allow the earlier diagnosis of chorioamnionitis. Most of these studies have analyzed the results of amniotic fluid immunological markers from amniotic fluid obtained by amniocentesis [11,12,13]. Amniocentesis has been shown to be a safe procedure to collect amniotic fluid [14], but it is more complicated in cases with PPROM due to a low amount of residual amniotic fluid. To our opinion, more effort should be made to develop noninvasive sampling techniques and to find the optimum cut-off values of biomarkers to aid in chorioamnionitis diagnosis for patients with PPROM. 

The aim of this study was to evaluate the interleukin-6 (IL-6), tumor necrosis factor alpha (TNF-α), and matrix metalloproteinase 8 (MMP-8) levels in vaginally obtained amniotic fluid to investigate their prognostic value and determine the most appropriate cut-off values for the prediction of histological chorioamnionitis. 

## 2. Materials and Methods

### 2.1. Patients and Samples

All patients who were admitted to Vilnius University Hospital Santaros Klinikos with diagnosed preterm premature ruptures of membranes before 34 weeks of gestation were invited to participate in this prospective case control study. In total, 185 participants who met the inclusion and exclusion criteria were included into the study. All participants signed informed consent form before enrolment. This study took place between July 2017 and July 2019 and was approved by the Vilnius Regional Biomedical Research Ethics Committee (2017-07-04 No. 158200-17-931-434). 

The inclusion criteria were the following: (1) maternal age ≥18 years, (2) singleton gestation, (3) gestational age 22+0–34+6 weeks, and (4) diagnosis of PPROM. Exclusion criteria included (1) multiple gestations, (2) fetal malformations, (3) vaginal bleeding, (4) placenta previa, and (5) nonreassuring fetal status. Calculation of gestational age was based on the last menstrual period and confirmed or modified by ultrasound scan at 11+0–13+6 weeks of gestation. Amniotic fluid leakage was diagnosed via examination with a sterile speculum to verify the pooling of amniotic fluid in the vagina. In the case of clinical doubt, amniotic fluid leaking was confirmed by the presence of the placental alpha microglobulin-1 protein in the cervicovaginal fluid (AmniSure^®^). According to our institution’s standard protocol, all women were managed expectantly with antibiotics and a single course of antenatal corticosteroids. Intravenous ampicillin 2 g and oral erythromycin 250 mg every 6 h were used for 48 h. The patients were then placed on oral amoxicillin 500 mg every 8 h and erythromycin 250 mg every 6 h to complete a 7-day course of antibiotic therapy. Clinical chorioamnionitis was defined following the Gibbs criteria [5].

Free-leaking amniotic fluid was obtained vaginally with a sterile speculum. Samples were collected every second day, centrifuged at 3000 rpm for 5 min at 4 °C, and stored at –80 °C. Samples containing obvious mucus, blood, and too-little volume were excluded from the study. Amniotic fluid samples from 156 participants who delivered within 48 h following amniotic fluid collection were included into the final analysis. Immunological assays of amniotic fluid samples were performed by the Enzyme Linked Immunosorbent Assay (ELISA) with commercial ELISA kits (Bender MedSystems, Vienna, Austria). For analysis by ELISA assay, nondiluted samples were used for the determination of IL-6 and TNF-α concentrations, and samples were diluted to 1:10 for the analysis of MMP-8. If measured concentrations of analytes exceeded the highest point on the standard curve, dilutions of 1:2, 1:5, or 1:10 were performed. Diluents were provided by the manufacturer. The concentrations of cytokines were calculated according to standard curves by a special program for the evaluation of ELISA results: Gen5 Microplate Data Collection & Analysis Software (BioTek Instruments, Winooski, VT, USA).

According to institution’s standard protocol, all postpartum placentas after preterm delivery were examined histologically. Chorioamnionitis was defined as the infiltration of the amnion, chorion, and parietal decidua with maternal neutrophils. Based on a histological analysis, patients were grouped into the histological chorioamnionitis group (Group I) or the group without diagnosed histological chorioamnionitis (Group II). All maternal and neonatal medical records were reviewed, and perinatal outcomes were recorded.

### 2.2. Statistical Analysis

Statistical analysis was performed using R package (version 4.0.3) (R Core Team, 2020). The distribution of the data was determined by the Shapiro–Wilk test. A comparison of means between the two groups was done using *t*-tests in cases where the data fit a normal distribution and the Mann–Whitney *U* test when the data did not fit a normal distribution. A Pearson chi-square test was used to determine differences between groups for categorical variables. Parametric continuous variables are expressed as means with standard deviations, nonparametric as median along with interquartile range (IQR), and categorical variables are presented as frequencies and percentages. The receiver operating characteristic curves were constructed to estimate the ability of variables to differentiate between groups. DeLong test was used to compare the areas under the curves (AUCs) of the different models. The best cut-off values to predict the outcome were determined by the Youden index. A *p*-value of <0.05 was considered statistically significant for all tests. 

## 3. Results

The study included 156 patients with a preterm premature rupture of the membrane before 34 weeks of gestation: 65 patients in Group I and 91 patients in Group II. The clinical characteristics of the patients and their distribution within the groups are listed in Table 1. The maternal age, gravidity, parity, comorbidities (gestational diabetes and hypertensive disorders), gestational age, latency between PROM and delivery, and neonatal birthweight were similar between groups and did not differ statistically. The colonization rate of Group B *Streptococcus* was higher and newborns had worse outcomes (lower Apgar score and umbilical pH) in Group I, where chorioamnionitis was diagnosed (*p*-value of <0.05). Clinical chorioamnionitis was diagnosed for six (9.2%) participants in group I and one (1%) participant in group II. 

**Figure 1:**
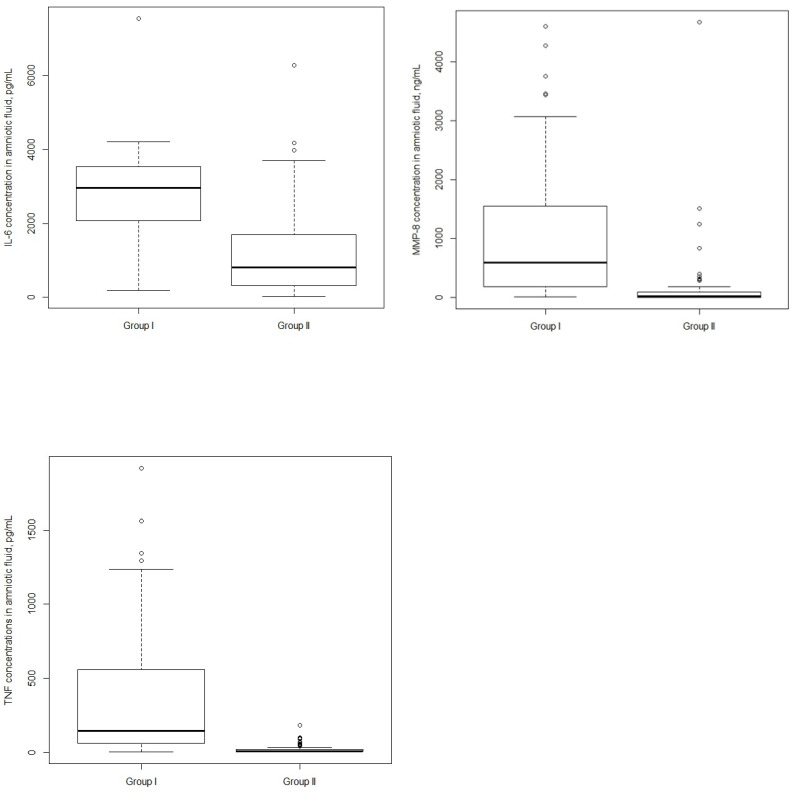
Interleukin-6 (IL-6), matrix metalloproteinase 8 (MMP-8), and tumor necrosis factor alpha (TNF-α) concentrations in amniotic fluid. Women with the presence of histological chorioamnionitishad a higher median of IL-6, MMP-8, and TNF- α concentrations than women without histological chorioamnionitis. *p*-value < 0.001 for all 3 assays. Horizontal bars indicate the median values. Group I, patients with diagnosed histological chorioamnionitis and Group II, patients without diagnosed histological chorioamnionitis.

The levels of IL-6, MMP-8, and TNF-α were statistically significantly higher in Group I than in Group II: median 2983.3 pg/mL (interquartile range or IQR: 2067.05–3881.69) vs. median 904.07 pg/mL (IQR: 325.27–1888.53), median 636.43 ng/mL (IQR: 190.82–1568.53) vs. 18.68 ng/mL (IQR: 4.55–93.27), and median 124.19 pg/mL (IQR: 63.84–595.97) vs. 8.64 pg/mL (IQR: 6.02–17.89), respectively (Figure 1).

**Figure 2:**
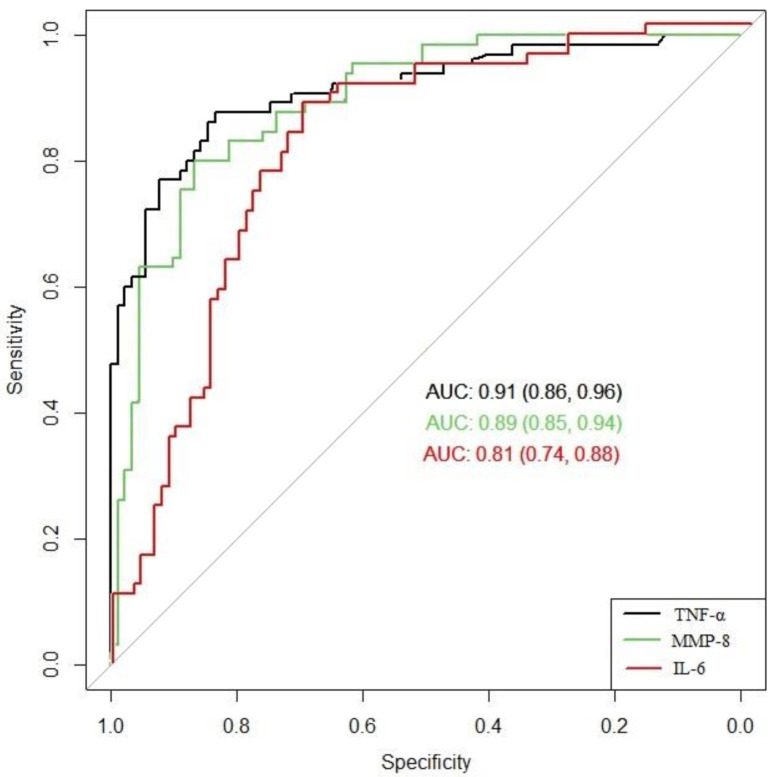
Comparison of the receiver operating characteristic (ROC) curves of IL-6, MMP-8, and TNF-α. AUC, area under the curve.

The ROC curves were constructed to determine the ability of IL-6, MMP-8, and TNF-α to differentiate between groups. Figure 2 shows a comparison of the ROC curves of these three immunological markers for the prediction of histological chorioamnionitis. TNF-α and MMP-8 predicted chorioamnionitis with AUCs of 0.91 and 0.89, respectively, while IL-6 predicted chorioamnionitis with an AUC of 0.81. The DeLong test demonstrated that TNF-α and MMP-8 have significantly different AUCs from IL-6, with a *p*-value < 0.05. No statistically significant difference was found for the comparison between the ROC curves of TNF-α and MMP-8 (*p*-value = 0.5).

The optimum cut-off values for IL-6, TNF-α, and MMP-8 were identified using the Youden index. To predict chorioamnionitis, the cut-off value for IL-6 was found to be 1389.82 pg/mL, and the sensitivity, specificity, PPV, and NPV were 88%, 70%, 67%, and 89%, respectively. For the same prediction, when the TNF-α cut-off value was 21.17 pg/mL, the sensitivity, specificity, PPV, and NPV were 88%, 84%, 79%, and 90%, respectively. The optimum cut-off value for MMP-8 was 172.53 ng/mL, and the sensitivity, specificity, PPV, and NPV were 80%, 87%, 81%, and 86%, respectively (Table 2). 

## 4. Discussion

Microbial invasion of the amniotic cavity induces a local inflammatory response, and this leads to increases in the concentrations of proinflammatory cytokines. Nowadays, a lot of attention has been given to the identification of changes in these immunological markers in amniotic fluid, as this could help to predict chorioamnionitis earlier. To our knowledge, this is one of the largest reported studies that has sought to determine cut-off levels for IL-6 and TNF-α, and it is the first study to determine the cut-off level for MMP-8 in vaginally obtained amniotic fluid for the prediction of chorioamnionitis. 

IL-6 is produced by monocytes and macrophages and is one of the first cytokines to be released in response to an infectious stimulus [15]. Meanwhile, TNF-α is an inflammatory cytokine that plays a role in the initiation of labor. The presence of an intrauterine infection induces the production of TNF-α in the amniotic fluid, and, therefore, TNF-α has been implicated in the pathogenesis of infection-associated preterm labor [16]. In this study, we found that women with chorioamnionitis had higher vaginal fluid IL-6 and TNF-α concentrations compared to women without this infection. In our study, the cut-off values of 1389.82 pg/mL for IL-6 and 21.17 pg/mL for TNF-α had high diagnostic values. Kacerovsky and colleagues also found that a cut-off value of 2500 pg/mL for IL-6 in vaginal fluid gave particularly good sensitivity, specificity, and negative predictive values for the identification of intra-amniotic inflammation [17]. However, this study was performed on individuals who developed PPROM at between 34 and 37 weeks of gestation. Another trial, which included women who developed PPROM at between 23 and 33 weeks of gestation, demonstrated high prognostic values for vaginal fluid IL-6 and TNF-α. Additionally, they found optimum cut-off values of 1000 pg/mL for IL-6 and 300 pg/mL for TNF-α [18]. Their study included 99 participants, and the amniotic fluid was collected by squeezing the vaginal secretions out of sanitary napkins with a commercially available garlic press. Kayem et al. found weak associations between vaginal IL-6 and TNF-α and maternal–fetal infections [19]. A controversial component of this study was that vaginal samples were taken twice weekly until delivery. So, the amniotic fluid collection-to-delivery interval remained unknown. This prevented us from analyzing whether the vaginal fluid IL-6 and TNF-α concentrations changed on the last day before labor, because there is a higher risk for the development of chorioamnionitis when there is a longer latency period in the rupture of the membranes [20,21].

The MMPs are a family of zinc-dependent endopeptidases that are expressed in many inflammatory conditions. They play a major role in the breakdown and digestion of the extracellular matrix, and through this process, they have been implicated in the rupture of fetal membranes [22,23]. In our research, we found that women with chorioamnionitis had higher vaginal fluid MMP-8 concentrations than women without this complication, and the cut-off value of 172.53 ng/mL had a particularly good diagnostic value. Many other studies have also demonstrated that an increased level of MMP-8 in amniotic fluid is closely related to intra-amniotic infections, and concentrations of higher than 23 ng/mL have high prognostic values for intra-amniotic inflammations/infections [24,25,26,27]. However, all of these trials included patients who underwent an amniocentesis. In our study, we found that the MMP-8 test had better specificity than the IL-6 test for the detection of chorioamnionitis. Similar results were demonstrated in a trial performed by Chaemsaithong and colleagues [27]. Holmstrom et al. conducted a study to investigate whether an elevated MMP-8 concentration could be detected from cervical fluid samples and showed that the cervical MMP-8 concentration in woman with PPROM did not differ between those with and without a microbial invasion of the amniotic cavity [28]. However, this study included 25 cases with PPROM, and only 10 of these cases were diagnosed with microbial invasion of the amniotic cavity.

Our findings, as well as those presented in previous reports, support the use of IL-6, TNF-α, and MMP-8 analyses for the identification of chorioamnionitis in patients presenting with PPROM. Our clinical expectation for the analysis of vaginally obtained amniotic fluid was that the procedure would be noninvasive, easily performed, and not associated with any complications. The MMP-8 and TNF-α analyses can improve the prediction of chorioamnionitis, which is a contraindication to the expectant management and can lead to expedited delivery.

### Strengths and Limitations

The strength of this study is that this is one of the largest reported studies that has sought to determine the cut-off levels for IL-6 and TNF-α, and it is the first study to determine a cut-off level for MMP-8 in vaginally obtained amniotic fluid for the prediction of chorioamnionitis. Additionally, all patients received standardized management with corticosteroids and antibiotics. The attending obstetrician-gynecologists had no knowledge of the patients’ cytokine levels, and, consequently, the patients with high cytokine levels did not undergo different management or earlier delivery.

The limitation of this study is that we used processed (centrifugated and frozen) amniotic fluid samples. However, Kacerovsky et al. demonstrated a strong correlation between the concentrations of immunological markers in fresh and processed amniotic fluid samples [29]. Additionally, vaginally obtained amniotic fluid samples were heterogenous with mucus and blood, which could have affected the immunological analyses. However, all the samples were obtained using the same technique, and the values of IL-6, TNF-α, and MMP-8 were statistically higher in the chorioamnionitis group than in the noninfection group. Moreover, chorioamnionitis was defined by histological examination of the placenta. Although histological chorioamnionitis is the gold standard for diagnosing intrauterine infections [9], there is controversy as to whether histological chorioamnionitis is correlated with higher rates of neonatal morbidity and mortality [9,30,31]. 

## 5. Conclusions

Vaginally obtained amniotic fluids with IL-6, MMP-8, and TNF-α seem to be good predictors for chorioamnionitis in patients with preterm premature rupture of the membranes before 34 weeks of gestation. The noninvasive technique of sampling amniotic fluid could be an alternative method to invasive amniocentesis. Further studies are required to identify the correlations between neonatal outcomes and concentrations of IL-6, TNF-α, and MMP-8 in vaginally obtained amniotic fluid. 

## Figures and Tables

**Table 1:** Characteristics of patients according to the groups. PPROM, preterm premature rupture of membranes, Group I, patients with diagnosed histological chorioamnionitis, and Group II, patients without diagnosed histological chorioamnionitis.

Characteristics	Group I (*n* = 65)	Group II (*n* = 91)	*p*-Value
Age of mother (years)	30.6 ± 6.4	31.2 ± 5.8	0.53
Primiparous, *n* (%)	32 (49.2)	46 (50.6)	0.87
Multiparous, *n* (%)	33 (50.8)	45 (49.4)	0.87
Primigravida, *n* (%)	27 (41.5)	34 (37.4)	0.59
Multigravida, *n* (%)	38 (58.5)	57 (62.6)	0.59
Gestational age at birth (weeks)	32^+0^ (27^+2^–33^+0^)	33^+0^ (28^+1^–33^+5^)	0.13
Latency between PPROM and delivery (hours)	43 (26–93)	41 (20–78)	0.29
Clinical chorioamnionitis, *n* (%)	6 (9.2)	1 (1%)	0.002
Birthweight (g)	1768.2 ± 669.1	1872.7 ± 603.2	0.51
Apgar score <7 at 5 min, *n* (%)	6 (9.2)	1 (1.1)	0.02
Umbilical cord arterial pH	7.31 ± 0.09	7.35 ± 0.09	0.003
Group B Streptococcus, *n* (%)	24 (36.9)	15 (16.5)	0.003
Gestational diabetes, *n* (%)	16 (17.6)	11 (16.9)	0.91
Hypertensive disorders, *n* (%)	12 (13.2)	8 (12.3)	0.87

**Table 2:** Cut-off levels for the sensitivity, specificity, and positive and negative predictive values of IL-6, TNF-α, and MMP-8. CI, confidence interval.

Cut-off Values	Sensitivity % (95% CI)	Specificity % (95% CI)	Positive Predictive Value % (95% CI)	Negative Predictive Value % (95% CI)
**IL-6 (pg/mL)**				
1389.82	88 (77–94)	70 (60–79)	67 (56–77)	89 (79–95)
2844.43	57 (44–69)	85 (76–91)	73 (58–84)	73 (64–81)
3427.52	37 (25–50)	90 (82–95)	73 (54–87)	67 (58–75)
4195.68	17 (9–28)	96 (89–99)	73 (45–92)	62 (53–70)
**MMP-8 (ng/mL)**				
413.67	63 (50–75)	96 (89–99)	91 (79–98)	78 (70–86)
294.36	65 (52–76)	90 (82–95)	82 (69–92)	78 (69–86)
172.53	80 (68–89)	87 (78–93)	81 (70–90)	86 (77–92)
124.5	83 (72–91)	81 (72–89)	76 (64–85)	87 (78–93)
**TNF-α (pg/mL)**				
70.77	72 (60–83)	95 (88–98)	90 (79–97)	83 (74–89)
50.39	78 (67–88)	89 (81–95)	84 (72–92)	85 (77–92)
24.56	86 (75–93)	85 (76–91)	80 (69–89)	90 (81–95)
21.17	88 (77–95)	84 (74–90)	79 (68–88)	90 (82–96)
